# Water Cure—Confessions and Observations of Sir Edward Lytton Bulwer

**Published:** 1846-01-01

**Authors:** 


### EDITORIAL, MEDICAL INTELLIGENCE, &c.

*Water - Cure.—Confessions and observations of Sir Edward Lytton Bulwer.*—We were beguiled into the purchase of a little treatise on the water-cure, by an announcement in the title page, that it was illustrated with the confessions of Sir Edward Lytton Bulwer. We were somewhat curious to read what a writer so distinguished for imaginative genius, poetic fervor, and eloquence of diction, would find worthy of his pen in the sanative attributes of water. We were disappointed, however, to discover that these "illustrations" constitute a very few pages only of the work, the remainder professing to develope the "philosophy of the water-cure, and the true principles of health and longevity."

It is a reprinted London publication. With the body of the work we have nothing to do. The writer is evidently a medical man, and we will do him the justice to say that he displays a considerable degree of familiarity with medical authors. The style is also lively, and not without piquancy. We more than suspect, however, that the work was done as a job to suit a publisher's purpose, and we should not be surprised if the name of John Balbernie, M. D., which appears on the title page, represents a man of straw. A large portion of the ground work of the 'philosophy,' is taken from Liebig's animal chemistry, without very particular acknowledgment. The medical profession comes in, of course, for the stereotyped charges, which, although hackneyed, are nevertheless very efficient arguments in behalf of any new form of quackery.

The confessions of Bulwer claim more consideration, inasmuch as we are quite willing to believe that he is honest in his declarations. We are willing to admit that in selecting a public newspaper for his confessional, he was not actuated solely by that egotistical desire, sometimes witnessed, to be on close terms of intimacy with the public, and have one's name a constant theme of conversation; but that his confessions were dictated by philanthropy, or a sense of duty. We also see no reason to doubt that he has really experienced much advantage from adopting this system.—"Then you subscribe to the efficacy of water as a panacea for all the maladies which flesh is heir to," exclaims the non-medical reader. To the medical reader, it is needless to say that this is not a legitimate conclusion from our admissions. A candid medical analysis of the 'confessions' renders it quite unnecessary, in order to have a rational explanation of the merits of the case, either to disprove the motives of the writer, or to deny the facts which are submitted. From the history itself, there is not the least difficulty in obtaining satisfactory insight into the philosophy of the magical effects which are there attributed to the influence of water; and it will be obvious to every medical reader of common observation, that medical science is in no wise compromised by the free admission that these effects actually occurred, making, of course, due allowance for the exaggerations incident to that fervent intensity, and hyperbolic quality of style which characterize the writings of Bulwer.

Divesting the narrative of all that is purely rhetorical, the following is a brief synopsis: Long continued intellectual labors, excitement and mental anxieties had exhausted the physical energies of a constitution naturally delicate, and especially had occasioned extreme disorder of the nervous system. A variety of functional derangements, of course, were incident to this general morbid condition. He had applied to various Physicians, who, he confesses, relieved his particular difficulties, "patched him up," as he expresses it. They, however, it is to be inferred, enjoined, in addition to such medicinal remedies as the particular symptoms indicated, the cessation of literary application, regulated diet and regimen, the recreation of travel, &c. These measures he had tried to put into practice; he does not specify how thoroughly, and forgets to mention how long his efforts were continued. One thing of importance he incidentally states, viz: he did not give up the use of wine. At length, tired of remedies prescribed by different advisers, and discouraged, he hears of the water-cure. He becomes impressed with the belief that the balm of Gilead is to be found at a Hydropathic establishment. The discouragement of his physicians and friends, only serve to strengthen this conviction. He resolves to go there: carries this resolution into effect; remains, in all, some seventeen weeks under its discipline; and at the expiration of this time he finds himself ronovated in body and mind to an astonishing degree. He expresses his sense of the agreeable change, with that ardor of enthusiasm, and eloquence of diction, which would be expected under such circomstances, from the pen of such a writer.

Now, viewing this history in a medical point of view, is there aught in it incomprehensible, or mysterious? Any physician who has had any experience in treating patients similarly affected, will assuredly read this narrative without amazement or incredulity. His regular medical advisers, we have every reason to presume, indicated a judicious course of treatment, which, had he properly pursued, would doubtless have succeeded to his most sanguine wishes. We infer from his own account that he did not thoroughly carry out the views of his physicians. Mental application, anxiety, fashionable dissipations, one, or all, doubtless continued to operate in perpetuating his ailments. He confesses that he continued the use of stimulants. He shifted from one medical adviser to another, evidently imbued with the common idea that he ought to have some prescription which would prove a specific remedy. In the latter, he was, of course, disappointed. The sore would not heal while the thorn remained. The trouble, in short, was, that his medical friends were unable to induce him to carry out the requisite sanative measures of diet, regimen, abstinence from literary labor, &c., which were necessary to his cure. Had it been otherwise, the same miraculous renovation would doubtless ensued—but would he in that case have come before the public in hyperbolical laudation of the means by which he had been renovated? We trow not. Hundreds of instances annually transpire under judicious medical advice of the faculty, but who thinks of publishing them to the world! We could not permit this to be done if our patients desired it; but quackery imposes no such restrictions.

It is not strange that the water-doctor, in this case, effected what the regular physicians failed to do. The latter, doubtless, after indicating the proper management to be pursued, left it for the patient to conform his conduct to it. In our instructions to patients, we often err in predicating too much on general mental superiority, forgetting that they are not only ignorant in matters pertaining to medicine, but quite as likely to entertain absurd notions on the subject of health, as those who are greatly below them in intellectual endowments. Whether this remark applies in the present instance, we are unable to say; it is fair to suspect that it may have been so. That quackery frequently carries with it vastly greater moral force than regular medical counsel, we have abundant occasion to observe. The fact that a man of intelligence is willing to place his life and health at its disposal, evinces a degree of confidence of which we cannot always avail ourselves. Of the value of faith in itself, we need not speak—but its greater value is in securing certain contingent observances which are the grand means relied upon for the result. In addition to this, from the moment that one occupying a conspicuous position commits himself to a form of quackery, the quack and himself enter into an involuntary partnership, with a mutual interest, in having the experiment succeed. The egotism of personal opinion, and the dread of ridicule if he is forced to acknowledge himself duped, conspire to create an intense anxiety of success, added to the already existing motives appertaining more directly to the recovery of health. These circumstances, doubtless, in the present instance, sufficed to accomplish what the mere instructions of the physician—addressed to the intellect alone, without any effort to excite the imagination, their execution left to himself, not carried out under his constant supervision—failed to accomplish. With a strong pre-conviction of success, an excited fancy, and every inducement to persevere in a faithful trial, he goes to the water establishment. Books and literary avocations are at length abandoned; physical exercise, plain diet, and regular habits adopted, and rigidly enforced; the wine bottle discarded; the various excitements and irregularities of fashionable life inaccessible,—it is surely not a very strange result that seventeen weeks of this sort of discipline were followed by most happy consequences.—Assuming that his ailments were not complicated with organic disease, they might very safely have been predicted. We have no desire to disparage the relative agency of the water-bath in the cure, and are willing to accord to it that invigorating effect for which it is often prescribed by the 'faculty,' without, however, vaunting it as panacea, or using it as a means of acting upon the credulity of our patients, and thereby cheating them into the performance of various conditions, upon which the chief dependence is placed. We are, also, far from feeling any dissatisfaction, from any professional feeling, with the success of the water-cure in this particular instance. Could the distinguished patient regard the matter in a truly philosophical view, he would perceive, that if cause for chagrin attaches to any, it is to himself, not to the medical profession. We sincerely trust that his health will be permanently restored, and that with the advantage of a more healthful physical condition, free from morbid influences, and artificial excitements, his future writings will have a purer moral tone, than has pervaded them hitherto. If the *mens sana in corpore sano* conduces to this result, we shall be still more gratified to join in congratulations on the success of his hydropathic experiment.

Our readers will perhaps think we have already extended our desultory remarks sufficiently for a topic of this nature; some reflections, however, have suggested themselves in reading these 'confessions,' to which we will devote a little space:

The medical observer cannot but be often amused, if not amazed at the egotism, and self-sufficiency, with which, of late, some individuals of literary celebrity, unfold to the public the details of their morbid experience, and pronounce thereupon medico-judicial decisions, intended to set aside as valueless all accumulated medical experience, and to render the aggregated learning and skill of the medical profession of no importance! If the public were to give heed to the advice of Bulwer, hydropathy would be deemed the great cure-all for bodily ailments. Miss Martineau feels it incumbent to expose her infirmities to the public, in order that those who are afflicted with disease may avail themselves of the never failing resources of mesmerism. Bryant comes before the public as a lecturer and ardent advocate of homœopathy—and so the list might be extended.

We are charitable enough to accord to these persons both honesty and philanthropy; but what would be incredibly wonderful if it were not so common, is, that they appear to predicate upon their general literary acquirements a seer-like capacity to deduce general principles of pathology and therapeutics from the phenomena of their consciousness, not deeming it of the least consideration whether they conflict with the opinions of those whose minds have been trained to philosophical investigations in this department of knowledge, and the business of whose lives is to develope or confirm medical truth! Every medical observer knows how deceptive are personal sensations, and the superficial aspects of single cases in estimating the correctness of a pathological principle, or the general applicability of any remedy. The physician has abundant occasion to learn the necessity of the utmost caution in deducing general principles from a limited series of facts. He well knows that a great number of contingent elements are more or less involved in every medical problem—in short, that numerous, great, and peculiar difficulties encompass the investigation of medical truth. We meet, also, with constant illustrations in our daily walks, of the extravagant errors which are made, as well among the educated and intelligent, as the ignorant and unlearned, respecting the efficacy of particular methods of treatment in their individual experience. It is needless to add, that certain preparatory studies are involved in medical reasoning, in addition to practiced habits of observation and ratiocination. All these conditions and qualifications are of no account in the apprehension of certain *literati*, who claim to expound medical truth, and to enunciate infallible rules of medical practice. We hope we are not deficient in our regard for literature, or in respect for those whose genius and taste entitle them to merited distinction, but as a medical man we cannot shut our eyes to the preposterous absurdity of supposing that because one is a successful writer of novels, or poetry, he is a whit better qualified to discriminate, or invested with authority to decide for the public in matters appertaining to physic. Extremes are often found to meet. In the often quoted saying of Napoleon, it is but a single step from the sublime to the ridiculous. The gifted and intellectual eulogists of quackery in its various forms, in this, as in some other things, are on the same platform with the most ignorant and credulous of society. They who are not distinguished excepting for good practical common sense, on this, as on other subjects, are generally the shrewdest, and most correct in their judgments. We deem this fact a valuable tribute to the character of medicine and the medical profession. The inconsistency of these literary crusaders against the faculty is striking. Let them be seized with severe illness, and safely carried through by the skilful management of a regular Physician, little is thought of it—at least not enough to write essays, lectures, or letters for publication on the subject. Orthodox practice cannot thus avail itself of the eclat of curing distinguished personages. But let some empirical absurdity come into vogue, and be fortunate enough to draw some literary person of eminence within its vortex, straightway, upon the four winds, bulletins are dispatched, in effect holding the following language: "*Eureka!* the philosopher's stone is at length discovered, since I have condescended to search for it!—Henceforth, let no one place any confidence in medical men, but follow my recommendation and example, in patronizing the newly discovered panacea, which promises to all, health and longevity!"

As a class of men practicing a calling, which, as it seems to us, has some peculiar claims upon the respect and consideration of the public, we might naturally enough feel somewhat impatient of this injustice, and complain, with some bitterness of feeling, of those who bring the force of their talents and influence to lessen the popular estimation of our profession. We have, however, been too long accustomed to such things not to have acquired sufficient endurance, and we believe they are generally regarded by the profession with philosophical equinanimity. The satisfaction of exercising our power to do good, goes far toward compensating for the absence of that valuation of its beneficial results, which, it must be confessed, is always gratifying and encouraging. But, it is happily the case, that we may rely on the grateful and kindly appreciation of the larger portion of those who are the recipients of its benefits. We do not need assurances that medical science is in no danger from these or any sources of attack and opposition; or that it will fail to advance, as it has done, and is doing, enlarging constantly its capacity of benefitting mankind; nor have we any reason to doubt, that in proportion with its progress, it will implant itself more deeply and securely in popular estimation. If we were disposed to retaliate upon those to whom we have particularly referred in the foregoing remarks, we could not desire better retributive justice than the mortification which is likely to be self-inflicted by longer reflection and observation, or, perchance, personal experience. We do not find much occasion for annoyance in the spirit and tone of feeling toward our profession, in the "confessions" of Bulwer, but we doubt not, if he live a few years longer, he will feel abundantly ashamed of them, and regret his precipitancy in submitting them to the public eye.

